# Effectiveness evaluation of the school-based drug prevention program #Tamojunto2.0: protocol of a cluster randomized controlled trial

**DOI:** 10.1186/s12889-019-7090-9

**Published:** 2019-06-13

**Authors:** Zila M. Sanchez, Juliana Y. Valente, Ana Paula Dias Pereira, Hugo Cogo-Moreira, Márcia H. S. Melo, Sheila C. Caetano, Jair J. Mari

**Affiliations:** 1 Department of Preventive Medicine, Universidade Federal de São Paulo, Rua Botucatu, 740, São Paulo, 04023-062 Brazil; 2 Department of Psychiatry, Universidade Federal de São Paulo, Rua Major Maragliano, 241, São Paulo, 04017-030 Brazil; 3 Department of Clinical Psychology, Universidade Federal de São Paulo, Av. Professor Melo Moraes, 1721, São Paulo, 05508-030 Brazil

**Keywords:** Randomized controlled trial, Prevention programs, Drug use, Protocol study

## Abstract

**Background:**

The European school-based drug addiction prevention program Unplugged was adapted to the Brazilian context by the Ministry of Health and renamed #Tamojunto. Its first implementations, in the form of a public policy in Brazil, showed contradictory and different effects from those observed in Europe. Adaptations were made to #Tamojunto in 2018 to reintroduce the essential content of the original program.

**Methods:**

A parallel, two-arm, randomized controlled trial (RCT) will be conducted to evaluate the effectiveness of the new version of the school-based government program #Tamojunto2.0 for the prevention of drug use among 8th grade middle school students from 70 public schools in three Brazilian cities, totaling approximately 6.300 participating students distributed in 210 classes. For intervention, the experimental group will receive the 12 lessons of the #Tamojunto2.0 program under the supervision of a Brazilian Ministry of Health team. The control group will not receive any intervention. Information will be collected from the students at three time points: preintervention and 9 and 18 months postintervention. Multilevel analyses will be performed using the Gllamm Stata program to assess simultaneous differences in prevalence, in time and among groups for the outcomes of interest. Structural equation modeling will be used to evaluate the effectiveness of the intervention in changing the behavioral patterns of the adolescents through latent transition analysis. The effect of the mediators involved in the program effectiveness outcomes will also be analyzed. The program doses applied in all classes of the intervention group will be collected using a form completed by the teacher at the end of each lesson, indicating the activities taught and not taught in each lesson.

**Discussion:**

This study will show whether the #Tamojunto2.0 program can be expanded as a public policy for all schools with the aim of preventing drug use among Brazilian students.

**Trial registration:**

Brazilian Clinical Trials Registry (RBR-8cnkwq) under the name “Avaliação do Efeito do Programa de Prevenção Escolar ao Uso de Drogas #Tamojunto2.0, Versão 2018”, on August 30th, 2018 (http://www.ensaiosclinicos.gov.br/rg/RBR-8cnkwq/).

## Background

The abuse of alcohol and other drugs is currently a major public health issue [1], and adolescent substance use is considered one of the main risk behaviors for the development of subsequent problems, such as dependence [2], cognitive impairment [3] and psychiatric disorders [4]. In Brazil, 55.5% of students between the ages of 13 and 15 have reported the consumption of alcoholic beverages, and 9% have reported illicit drug use [5].

The high prevalence of drug use in adolescence and the early age of onset of this behavior show the need for prevention programs in these age groups [6, 7]. School-based prevention programs have been implemented worldwide to reduce or delay the onset of alcohol and other drug use among adolescents [8]. Among the prevention models that have been shown to be effective are those that consider the social influences on the initiation of consumption, working towards reducing the risk factors and increasing the protective factors [9–11].

In 2013, the Brazilian Ministry of Health (BMH), in partnership with the United Nations Office on Drugs and Crime (UNODC), carried out a cross-cultural adaptation of Unplugged—a school-based program for substance use prevention*,* which was called #Tamojunto in Brazil [12]. The program is based on the “Comprehensive Social Influence Model” [13], whose approach attempts to build specific skills in adolescents that enable them to manage social influences, deconstructing normative beliefs, and is based on reflections on the contexts of drug use and knowledge about drugs and their health effects. When evaluated in European countries, Unplugged produced significant reductions in reports of episodes of drunkenness, frequent use of marijuana [14], tobacco use and use of any drug [15].

After translation into Brazilian Portuguese and cultural adaptation, in 2014 and 2015, the first Brazilian study on the effectiveness of #Tamojunto was conducted through a randomized controlled trial in six cities involving a sample of 6391 students from 72 public schools. The short-term (nine month follow-up) [16] and long-term (21 month follow-up) results showed a negative effect on alcohol initiation [17]. The adolescents in the intervention group had a 30% higher risk (95%CI 1.13–1.49) at 9 months and a 13% higher risk (95%CI 1.01–1.27) [16] at 21 months for first alcohol use compared to those in the control group [17]. The main hypothesis to explain these iatrogenic results suggests inadequate cultural adaptations for the alcohol components. The adaptation did not include the original components that reinforced the nonuse of alcohol and emphasized only the prevention components aimed at avoiding alcohol intoxication, with an emphasis on harm reduction [18].

Considering the negative results of #Tamojunto, in 2018, there was a significant change in the activities and content of the lessons that addressed alcohol and its effects. This new adaptation of the material aims to reintroduce the central elements of the European Unplugged program [14] and now requires a new evaluation of its results in the Brazilian population before expanding it into a formal public policy. This new adaptation also comes with a change in its visual identity, and the name has been changed to #Tamojunto2.0.

It is important to emphasize that drug prevention programs based on a theoretical psychosocial framework usually have protective effects on other risk behaviors of adolescents [11], as they act on risk and protective factors shared by several negative health outcomes, such as violence and psychiatric disorders [19–21]. In this sense, it is also important to investigate the effects of the #Tamojunto2.0 program on secondary outcomes, such as violence and different psychiatric symptoms, in Brazil.

## Methods

### Aims

The main objective of the study is to evaluate the effectiveness of the school-based program #Tamojunto2.0 at 9 and 18 months of follow-up in the prevention of drug use (alcohol, tobacco, inhalants, marijuana, cocaine, crack and amphetamines) and binge drinking among Brazilian students. Our main hypothesis is that the #Tamojunto2.0 program will delay the first use of these drugs and will decrease the prevalence of use among students who participate in the intervention compared to the students in the control group.

The second objective of this study is to evaluate the effectiveness of the program at 9 and 18 months of follow-up in the prevention of school violence and psychiatric symptoms (depression, anxiety, *attention-deficit/hyperactivity disorder* and eating disorders) among Brazilian students. Our second hypothesis is that the #Tamojunto2.0 program will reduce the prevalence and incidence of secondary outcomes among students participating in the intervention compared to the students in the control group.

The third objective of the study is to evaluate the mediating effect of the skills taught by the program (decision-making skills, refusal skills, knowledge about drugs and beliefs about drugs) in reducing primary outcomes (alcohol, tobacco, marijuana, inhalant, cocaine, crack and amphetamine use and binge drinking) and secondary outcomes (reports of school violence and psychiatric symptoms), comparing the control and intervention groups at three different time points (0, 9 and 18 months). Our third hypothesis is that the improvement in the skills taught by the program will mediate the program’s effect on reducing the primary and secondary outcomes, as described by a logical model.

Finally, the fourth objective of the present study is to evaluate the program implementation fidelity to identify possible changes in the original content and to measure if the dose of the program offered to the students influences the effects of the program. Our fourth hypothesis is that the greater the dose of the program offered to the students and the smaller the changes to the original content are, the greater the effect of the program in the prevention of the proposed outcomes will be.

### Study design

A randomized controlled trial with two parallel groups (intervention and control) will be conducted to evaluate the effectiveness of the program for adolescents enrolled in 8th grade middle school in 70 public schools in three Brazilian cities, totaling approximately 6300 students distributed in 210 groups. Randomization will be conducted at the school level. Schools randomly selected to be part of the intervention group will offer the 12 lesson #Tamojunto2.0 program in the year 2019. Schools in the control group will not offer prevention programs or activities in 2019. The selection of schools and the allocation to the control or intervention group will be performed by a simple random draw from the list of the National Institute for Educational Studies and Research ‘Anísio Teixeira’ (INEP), which includes all schools from each municipality.

The baseline data (T1) will be collected two weeks before implementation of the program in February/March 2019, and the follow-up data will be collected at 9 months (T2) in November/December 2019 and at 18 months (T3), August/September 2020 after the initial data collection in August 2020. The data will be collected simultaneously for both groups. This protocol was prepared in accordance with SPIRIT 2013 [22], and the study will be conducted according to the CONSORT guidelines [23].

**Fig. 1:**
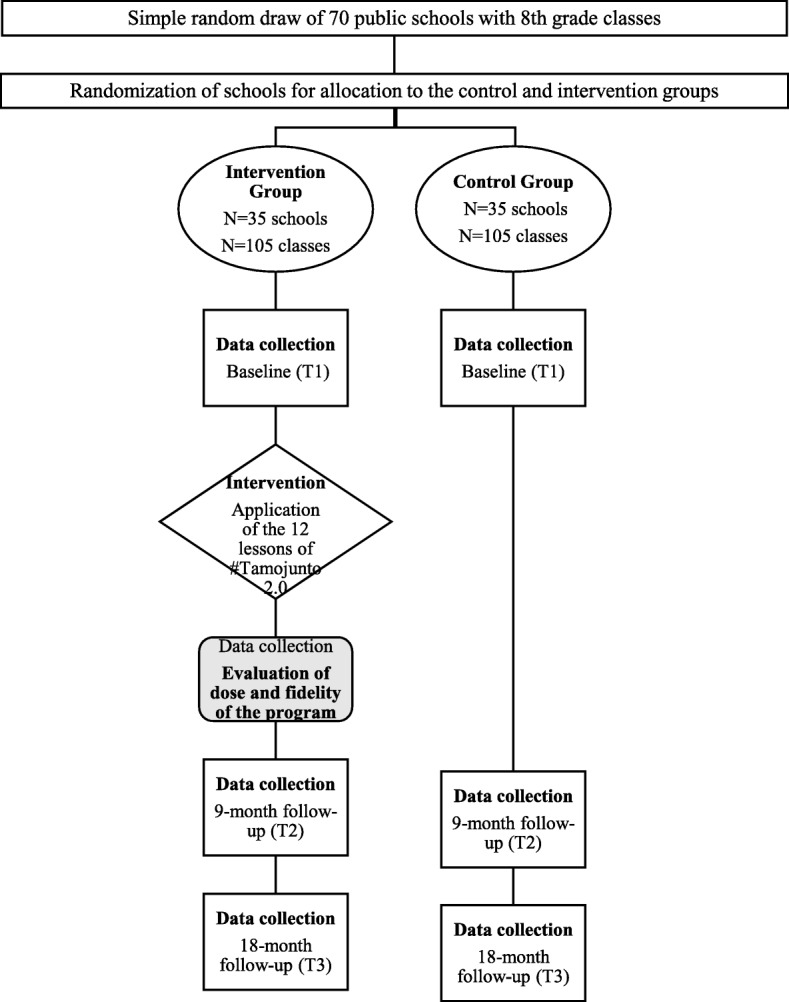
Flowchart of the general design of the effectiveness evaluation of Tamojunto 2.0

At the end of each lesson, all teachers in the experimental classes will complete a fidelity and dose form, stating which of the planned activities were or were not taught in the lesson and if the teacher made any changes. Figure 1 shows the study design flowchart.

### Participants

Eighth-grade middle school students aged 12 to 14 years, enrolled in public schools from three Brazilian cities (Fortaleza, Eusébio - northeastern region - and São Paulo - southeastern region) will participate in the effectiveness evaluation of the program. The participating cities were recommended by the BMH, based on the identification of the municipalities in which there was already collaboration and a partnership with the local government for the application of the #Tamojunto2.0 program*.* In addition, the selected cities have large sociodemographic differences; the two cities in the northeast are poorer and less populous than São Paulo, which is the fifth most populous city and the tenth richest city in the world.

### Sample size calculation

The PASS 15.0 software was used to calculate the sample sizes of the two intervention groups in a cluster randomized design [24]. A sample size of at least 3150 adolescents in the control group and 3150 in the intervention group, distributed among 35 clusters (schools) with at least 90 subjects in each arm, will reach a power of 82% in identifying a difference between groups of 2.5% for the outcome of binge drinking in the past month, with an initial prevalence of 10%, a significance level of 5%, and an intraclass correlation of 0.005. Thus, it is necessary to ensure the inclusion of at least three 8th grade classes from each sampled school, with 35 schools in the intervention group and 35 in the control group. Therefore, 70 schools will be selected to ensure a minimum sample size of 6300 adolescents, considering possible losses to follow-up.

### Randomization

Schools from each of the participating municipalities registered in the INEP’s national registry of schools will be randomly selected. The schools must meet the following inclusion criteria: (a) a public school and (b) have at least three 8th grade classes. Thirty schools will be drawn in Fortaleza, 30 schools in São Paulo and 10 schools in Eusébio. Among the schools selected to participate in the study, a second random draw will define which schools will be included in the control group and which will be included in the experimental group, maintaining a ratio of 1:1 in the number of control and experimental schools in each municipality. After selecting the schools, a list with the names of the selected schools will be forwarded to each education department of the participating municipalities to request authorization for participation in the study. Due to the involvement of the government, all schools typically agree to participate, as occurred in a previous study [16].

### Instrument

The instrument to be used for data collection in evaluating the program effectiveness was designed using instruments previously used in studies evaluating the effect of drug prevention programs both in Brazil [16, 25] and abroad [14]. One of the instruments used as a base was created by the EU-DAP (European Drug Addiction Prevention Trial) and used in a previous effectiveness evaluation of Unplugged [26]. In Brazil, this instrument has been translated and adapted into Portuguese [27], with some questions replaced by questions adopted from two questionnaires widely used in several studies among Brazilian students: the World Health Organization questionnaire used in the VI Brazilian Survey of Drug Use among Students [28] and the National Survey of School Health questionnaire used by the Ministry of Health [5]. Details of the instrument are presented in Table 1.

**Table 1:** Details of the instrument to be applied among the students at the evaluation time points

Variable	Main Use *	Source	Scale
Drug use in lifetime (first use, incidence and prevalence), past year and past month (prevalence)	Primary outcomes (alcohol, tobacco, marijuana, inhalant, cocaine, crack and amphetamine use and binge drinking)	CEBRID [28]	22 dichotomous questions
Socioeconomic class	Confounder	ABEP [35]	A scale with 15 items
Demographic data	Confounders	CEBRID [28]	2 questions (age and sex)
Decision-making	Mediator	EUDAP [26]	A scale with 9 dichotomous items
Positive and negative beliefs	Mediator		Two scales with 11 dichotomous items each (one for alcohol and one for marijuana)
Normative beliefs	Mediator		Two scales: a Likert scale with 4 items and a dichotomous scale with 11 items
Knowledge about drugs	Mediator		A scale with six items
Refusal skill	Mediator		A scale with three items
School violence	Secondary outcome	Olweus Bully Victim Questionnaire [30]	Two scales: a scale to measure violence perpetrated with eight dichotomous items each and a scale to measure violence suffered with seven dichotomous items each
Psychiatric symptoms	Secondary outcome	Strengths and Difficulties Questionnaire [31]	A scale with 25 dichotomous items
Eating disorders	Secondary outcome	SCOFF [33]	A scale with 5 dichotomous items

The form that evaluates dose and fidelity (Table 2), to be completed at the end of each lesson by the experimental group teachers, was prepared based on the EUDAP instrument [29].

**Table 2:** Instrument for evaluating the #Tamojunto2.0 program implementation dose and fidelity

Instrument	Completion	Goals	Content
Forms for monitoring the application of the #Tamojunto2.0 lesson-by-lesson. Teacher.	To be completed after each of the 12 program lessons by the teachers who will implement the program.	Assess the dose of program activities being provided by teachers to students and whether the teacher is following the original lesson plan of the program.	▪ The teacher will complete the form at the end of each class, reporting which of the activities planned for that lesson (as stated in the student and teacher handbooks) were actually fully taught to the students and whether any changes were made to the lessons.

To match the questionnaires across the evaluation time points, students will fill out a secret code on the first page of the questionnaire that involves generating letters and numbers from personal information. In this way, the codes can only be decoded by the students themselves. The codes allow the researchers to match individual questionnaires from different evaluation time points while providing the anonymity and confidentiality essential for a study of illicit behavior [36]. The databases of the two evaluation time points will be integrated by matching the secret codes using the Levenshtein algorithm, which can identify similarities between a set of characteristics [37].

### Primary outcomes

The primary outcomes will be assessed through dichotomous questions about use in the last 30 days (yes/no), in the last 12 months (yes/no), and prior to the study period (incidence = new cases) of the drugs alcohol, tobacco, marijuana, inhalants, cocaine, crack and amphetamines and binge drinking.

### Secondary outcomes

The following will be evaluated as secondary outcomes: 1. episodes of violence in school measured through two scales for evaluating bullying suffered and bullying perpetrated in the last 30 days [30] and 2. current psychiatric symptoms (anxiety, depression, hyperactivity and current behavior and symptoms of eating disorders) [31, 32] [33].

### Mediators

As mediators of program effectiveness, the following will be evaluated: 1. normative beliefs; 2. positive beliefs and negative beliefs about alcohol and marijuana use; 3. knowledge about drugs; 4. decision-making skills; and 5. refusal skills [34]. The scales for each mediator are described in Table 1.

In addition to the modules covering primary outcomes, secondary outcomes and mediators, the questionnaire also has a module on sociodemographic data. The socioeconomic class will be evaluated through the ABEP (Brazilian Market Research Association) scale [35] (Table 1**).**

### Intervention

In each of the experimental schools, all 8th grade students will receive the program #Tamojunto2.0, and the school will assign one teacher per class to receive training on the program.

The #Tamojunto2.0 program will be applied to students in the classroom by trained teachers. The 12 lessons, described in Table 3, use interactive methods and will be guided by student and teacher handbooks. On average, the lessons last 50 min each. The program consists of four lessons on attitudes and knowledge about drugs, four lessons on social and interpersonal skills, and four lessons on personal skills. Each lesson will have three to five activities addressing life skills [13, 38]. The teacher’s handbook provides information about each lesson’s procedures, objectives, required materials, activities to be followed and tips. Both handbooks are freely accessible and can be found in several languages at www.eudap.net.

**Table 3:** Description of the 12 lessons in the *#*Tamojunto 2.0 program, including their title, activities and goals

Lesson	Title	Activities	Goals
**1**	#Tamojunto introduction	▪ Presentation ▪ Group work: coexistence contract management ▪ Homework	Introduction to the program, establishment of rules for the lessons and reflection on what is known about drugs
**2**	Where do I belong?	▪ Situation play ▪ Game discussion	Clarify the influences and expectations of the group
**3**	Choices: protective and risk factors for alcohol use	▪ Information on different factors influencing drug use ▪ Discussion and work in small groups	Information on different factors influencing drug use
**4**	What do you think reflects reality?	▪ Presentation ▪ General discussion ▪ Group work ▪ Game	Encourage critical analysis of information, reflection on differences between personal opinions and actual data, reassessment of norms
**5**	What we know and do not know about smoking	▪ Quiz ▪ General discussion ▪ Feedback ▪ Game	Information on the effects of smoking, differentiation of expected vs. real effects and short-term vs. long-term effects
**6**	Express yourself	▪ Game ▪ Discussion and plenary ▪ Group work	Proper communication of emotions, distinction between verbal and nonverbal communication
**7**	Manifest yourself in the world and in your life	▪ General discussion ▪ Group work ▪ Role play	Promote assertiveness and respect for others
**8**	The new guy!	▪ Role play ▪ Game ▪ General discussion of the class	Recognition and appreciation of positive qualities, acceptance of positive feedback, practice and reflection on how to get to know new people
**9**	Drugs—educate yourself	▪ Group work ▪ Question and answer game	Information on the positive and negative effects of drug use
**10**	Coping strategies	▪ Presentation ▪ General discussion ▪ Group work	Expression of negative feelings, dealing with challenges
**11**	Problem solving and decision-making	▪ Presentation ▪ General discussion ▪ Group work ▪ Homework	Problem solving and fostering creative thinking and self-control
**12**	Setting goals and final considerations	▪ Game ▪ Group work ▪ General discussion	Distinguish the long-term and short-term goals, evaluate the program and its process

In Brazil, the English materials were translated into Portuguese, with adaptation of idiomatic expressions and the substitution of information about heroin for information about crack, as well as changes in the lessons addressing alcohol use [28]. The new adaptation, created in 2018, removed the changes made to the alcohol use lessons during the years 2014 and 2015 and changed class 3 on alcohol back to its original form (i.e., the European model) to remove the possible iatrogenic cultural adaptations regarding alcohol use [16]. Therefore, in this protocol, we describe a study that will be carried out in the years 2019 and 2020 to evaluate the revised version of the #Tamojunto program [16], now called #Tamojunto 2.0.

The teachers who will administer the program will be trained in a 16-h training program conducted by professionals from the BMH. In addition, the implementation will be accompanied by two on-site visits by BMH professionals throughout the 12 lessons and by a virtual forum where teachers can post questions about the application of the activities in the lessons.

### Data analysis of the randomized controlled trial

The data will initially be analyzed by a descriptive analysis, that is, qualitative variables will be summarized as numbers and percentages and numerical variables as means, standard deviations, medians, minima and maxima. Descriptive analyses will be performed for different patterns of drug use. This dataset will be used to calculate sample weights through the svy command in Stata so that the baseline data analysis can be presented in an expanded form, aiming at correcting for losses and extrapolating the data to the sample universe [39, 40].

All analyses will be carried out in the two samples: “intention to treat” (ITT), which will include all the participating classes that received at least one lesson of the program, and “per protocol” (PP). The PP analysis will include only those classes that receive the complete protocol (12 lessons), determined based on evaluating the dose and fidelity form that will be completed by the teacher at the end of each lesson.

Because the data are collected in groups, they are not independent at two levels (school and class), and the measures of association should be analyzed through GLLAMM (Generalized Linear Latent and Mixed Models), which calculates the level of dependence between the data [41]. Due to the hierarchical structure of the data, multilevel models will be used to simultaneously test for differences in the prevalence of outcomes between times and between groups, considering schools as a modeling level (Gllamm Stata 14). All analyses will be adjusted by sex, socioeconomic level and site. The analyses will be performed in Stata 15 (descriptive analyses and multilevel GLLAMM), and a significance level of 5% will be adopted.

**Fig. 2:**
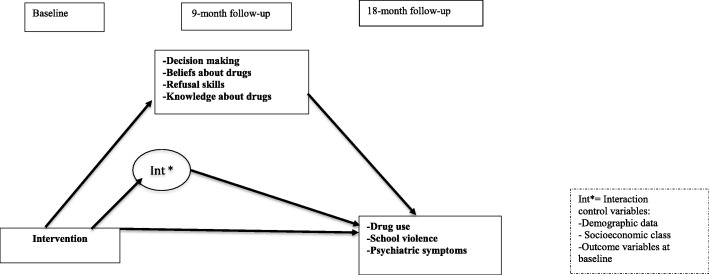
Mediation model for the outcomes of students participating in the evaluation of the #Tamojunto2.0. program; Three mediation models will be generated using the outcome variables of drug use in the past year and in the past month and incidence of drug use

Structural equation modeling (SEM) will be used to analyze the mediators involved in reducing the primary and secondary outcomes over 18 months [42] (Fig. 2) and to assess the effectiveness of the intervention in changing the behavioral patterns of adolescents related to drug use and violence through latent transition analysis (LTA). LTA can be understood as a longitudinal extension of latent class analysis, as it is used to identify and describe the ideal number of classes representing the study population, following their transition over a given period [43]. This approach offers great advantages compared to traditional logistic regression methods in longitudinal studies, such as a lower rate of false discovery compared to multilevel logistic regression models when assessing the effects of an intervention on multiple outcomes [44]. Drug use is often studied with the latent class model due to the high correlation observed between the different drugs used, addressing the heterogeneity of drug use behavior in adolescents [45]. These analyses will be performed with the Mplus 8 software, and a significance level of 5% will be adopted.

### Dose and Fidelity evaluation data analysis

Quantitative data will be analyzed using the Stata 14 software using descriptive statistics, considering frequencies and prevalence. Inferential statistics will be applied to test hypotheses of differences between observations and observers.

The qualitative data will be transcribed in full and categorized according to their content similarity. The computer software NVivo version 10 will be used for the qualitative analysis [46].

## Trial status

By the time of this submission (February/2019), the study is in the recruitment and agreement phase with the selected schools.

## Discussion

This study protocol describes a randomized controlled trial to evaluate the effectiveness of the school-based drug prevention program #Tamojunto 2.0. The main objective of this study is to evaluate the effectiveness of the intervention in terms of drug use outcomes among Brazilian students. Data on school violence, psychiatric symptoms, and mediators of the program’s effectiveness, such as decision-making skills, refusal skills and knowledge and beliefs about drugs, will also be collected.

### Strengths and limitations

One of the strengths of this study is that it will include a representative sample of students from Brazilian public schools (*n* = 6300), obtained through a simple random draw of schools from the universe of schools in each municipality. In addition, this study will have three data collection points over time and can thus assess possible short-term and long-term effects of the program and enable more sophisticated analyses, such as mediation analysis and latent class transition analysis. Third, the study will use a school randomization design, which minimizes the effects of contamination that can occur in a project that randomizes the classes within each school. An additional strength of the study is that—in contrast to most RCT studies—we will focus not only on the effectiveness of the program but also on the mediators of change, which will shed light on how the intervention works and enable the proposal of possible mechanisms of action, as has already occurred in a pilot manner by the research group itself [16].

To contribute to the understanding of the intervention results, the present study will examine whether the intervention was implemented according to the original plan and the dose received in each participating class through the use of a dose and fidelity evaluation. The dose received in the implementation and the fidelity (i.e., how closely the program follows the original curriculum) are elements related to the expected outcomes of the program [47]. In this way, the study will identify the real conditions of the program implementation in the Brazilian public education system. Given the results obtained by Sanchez et al. [16], showing that the first version of the adaptation of the Unplugged program in Brazil resulted in a 30% increase in alcohol initiation by students, and considering the important changes made to the material to reverse this effect, it is important to evaluate whether 1) removing the components with a possible iatrogenic effect from the alcohol lesson was truly effective and 2) whether the lessons are being taught exactly as described in the handbooks provided to the teachers and students.

Some limitations of this study must be noted. The main limitation is related to the known high rate of school absences of the Brazilian student population, which could result in bias. However, this is a common limitation to longitudinal studies [48–50], and the goal is to try to minimize it by giving prior notice to the students about the researchers’ visit for data collection, which did not occur in previous versions of the study [16]. Another limitation of the study is that the information is obtained through self-reporting by the students themselves. Although this method may lead to measurement bias, self-reporting is the most recommended evaluation method for adolescents and has been found to have excellent validity in studies evaluating drug-related behaviors [51, 52]. Currently, there are no viable alternatives for collecting data on alcohol use in adolescent samples, as biological measures would not be appropriate in a sample in the early stages of alcohol use [53]. To avoid the overreporting of drug use, a question of the use of fictitious drugs (holoten and carpinol) has been added to the questionnaire to screen this problem. In cases where the student responds positively to these drugs, the questionnaire will be excluded from the study, reducing the information bias [54].

## Conclusion

Considering the iatrogenic results of the first version of #Tamojunto, a new adaptation of the material was carried out to better reflect the original content of Unplugged, which has already shown positive preventive results in the European context. Thus, a randomized controlled trial protocol with the inclusion of implementation dose and fidelity measures was designed to evaluate the effectiveness of the new version of the program that will be applied by the Brazilian government, now titled #Tamojunto 2.0. Considering that the #Tamojunto 2.0. program was developed with the aim of being applied as a universal intervention, within a context of a national public policy, we believe that this study will contribute to supporting decision-making regarding the dissemination of the new version of this program in Brazilian public schools.

## Data Availability

Not applicable.

## Abbreviations

ABEP: Brazilian Market Research Association
BMH: Brazilian Ministry of Health
EU-DAP: European Drug Addiction Prevention Trial
GLLAMM: Generalized Linear Latent and Mixed Models
INEP: National Institute for Educational Studies and Research ‘Anísio Teixeira’
ITT: Intention To Treat
LTA: Latent Transition Analysis
PP: Per Protocol
RCT: randomized controlled trial
SEM: Structural equation modeling
UNODC: United Nations Office on Drugs and Crime
